# Valve-in-Ring TMVR Following Antegrade Base-to-Tip LAMPOON With ELASTIC on VA-ECMO Support: A Case Report

**DOI:** 10.1016/j.jscai.2026.105394

**Published:** 2026-05-14

**Authors:** Chantal Y. Asselin, Mostafa Naguib, Christopher Song, Konstantinos Koulogiannis, Robert M. Kipperman, Howard Axelrod, Linda D. Gillam, Philippe Généreux, Gennaro Giustino

**Affiliations:** Gagnon Cardiovascular Institute, Atlantic Health System, Morristown, New Jersey

**Keywords:** case report, electrosurgical laceration of Alfieri stitch, laceration of the anterior mitral valve leaflet to prevent outflow obstruction, left ventricular outflow tract, transcatheter mitral valve replacement

## Abstract

Transcatheter mitral valve replacement (TMVR) in failed surgical rings with an Alfieri stich represents a therapeutic challenge. A 70-year-old man with prior robotic surgical mitral valve repair with an incomplete ring and an Alfieri stich presented with acute decompensated heart failure and was found to have severe mitral regurgitation and pulmonary hypertension. He was deemed not to be suitable for redo surgery. Preprocedural computed tomography demonstrated anterior mitral leaflet length of 29 mm, indicative of high-risk for dynamic left ventricular outflow tract obstruction. This case described the successful combination of antegrade laceration of the anterior mitral valve leaflet to prevent outflow obstruction and electrosurgical laceration of Alfieri stitch to facilitate valve-in-ring TMVR while on venoarterial extracorporeal membrane oxygenation support to prevent hemodynamic compromise during high-risk valve-in-ring TMVR.

Left ventricular outflow tract obstruction (LVOTO) is a devastating complication of transcatheter mitral valve replacement (TMVR). Historically, the risk of LVOTO in prohibitive-risk surgical patients precluded transcatheter heart valve replacement. Furthermore, existence of an Alfieri stich complicates TMVR. Electrosurgical techniques have emerged as a therapeutic option in these complex patients. Laceration of the anterior mitral valve leaflet to prevent outflow obstruction (LAMPOON), is an electrosurgical leaflet modification technique, which can prevent LVOTO.^1^ Electrosurgical laceration of Alfieri stitch (ELASTIC) can lacerate the pre-existing Alfieri stitch.^2^ The combined use of these techniques using venoarterial (VA) extracorporeal membrane oxygenation (ECMO) has not been described before.

A 70-year-old man underwent robotic mitral valve repair with a 30-mm incomplete Future Band (Medtronic) and A2-P2 Alfieri stitch 2 years prior at an outside hospital. He presented with acute decompensated heart failure and was found to have low-flow low-gradient severe aortic stenosis and combined severe mitral regurgitation and stenosis. He underwent transcatheter aortic valve replacement with a 29-mm Evolut (Medtronic) implanted deep to facilitate future TMVR in the incomplete ring. He then re-presented 6 months later with heart failure, severe pulmonary hypertension (pulmonary artery systolic pressure of 80 mm Hg), and right ventricular dysfunction. Preprocedural computed tomography demonstrated an Emory angle of 9°, anterior mitral leaflet (AML) length of 29 mm predisposing to leaflet overhang, and high-risk of dynamic LVOTO (observed when the AML length was >24 mm) (Figure 1). He was deemed at prohibitive surgical risk due to the presence of multiple comorbidities, including severe pulmonary hypertension, right ventricular dysfunction, and cardiogenic liver cirrhosis.

**Figure 1:**
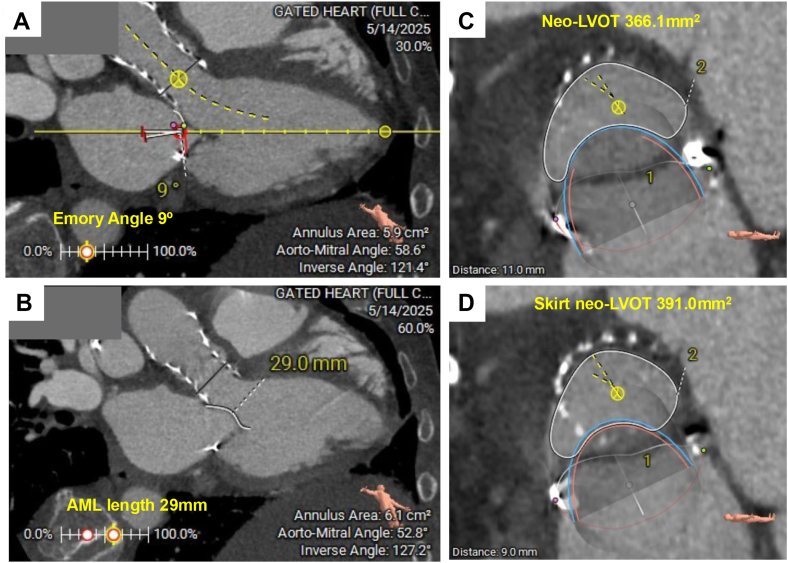
**Preprocedural computed tomography.** (**A**) Emory angle 9°. (**B**) AML length of 29 mm (**C**) Neo-LVOT of 366.1 mm^2^. (**D**) Skirt neo-LVOT of 391.0 mm^2^. AML, anterior mitral leaflet; LVOT, left ventricular outflow tract.

The patient was planned for simultaneous antegrade LAMPOON and ELASTIC of the Alfieri stich for complex valve-in-ring TMVR. Owing to the poor hemodynamic status, the patient was placed on VA-ECMO, expecting that the laceration of the Alfieri stitch and AML would worsen mitral regurgitation, leading to hemodynamic collapse due to the degree of pulmonary hypertension at baseline. After transseptal puncture, 2 Agilis guide wires (Abbott) were placed in the left atrium and an En-Snare in the left ventricular outflow tract within a medium-curve Agilis catheter and a 6F JR4 guide positioned across the medial orifice of the mitral valve. The base of the AML was successfully traversed with an electrified Astato 0.014-inch wire (Asahi Intecc) at 50 W cut mode, within an IM 6F guide (Cordis) and a steerable Versacross (Boston Scientific). After snaring, a “flying V” was created and positioned across the AML. Then, the wire was electrified, and simultaneous electrosurgical laceration of the AML and Alfieri stitch were performed in a 2-step traction (first to complete the AML laceration and second to lacerate the Alfieri stich) (Figure 2). The patient remained hemodynamically stable while on VA-ECMO support. An S3 29-mm valve (Edwards Lifesciences) was then implanted with >3 mL extra volume and prolonged pacing, followed by postdilation with ventricular flaring (Figure 3; Supplemental Video 1). Final transesophageal echocardiogram demonstrated excellent results (Figure 4; Supplemental Video 2). VA-ECMO was converted to left atrial VA-ECMO to further decongest the patient, lower intrapulmonary pressures, and optimize for extubation. The patient was decannulated on postoperative day 1, and the residual atrial septal defect was closed with an atrial septal occluder. At the 6-month follow-up, the patient was asymptomatic in New York Heart Association class I functional status.

**Figure 2:**
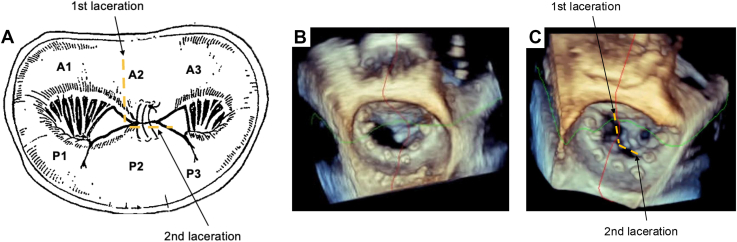
**Simultaneous electrosurgical laceration of the AML and Alfieri stitch using a 2-step traction technique.** (**A**) Schematic of 2-step traction set up with initial AML traversal and laceration track (orange line). (**B**) Pre-electrosurgery. (**C**) Postcut with laceration track (orange line). AML, anterior mitral leaflet; LVOT, left ventricular outflow tract. AML, anterior mitral leaflet; ECMO, extracorporeal membrane oxygenation; ELASTIC, electrosurgical laceration of Alfieri stitch; LAMPOON, laceration of the anterior mitral valve leaflet to prevent outflow obstruction; LAVA, left atrial venoarterial; TEE, transesophageal echocardiogram; TMVR, transcatheter mitral valve replacement; VA, venoarterial.

**Figure 3:**
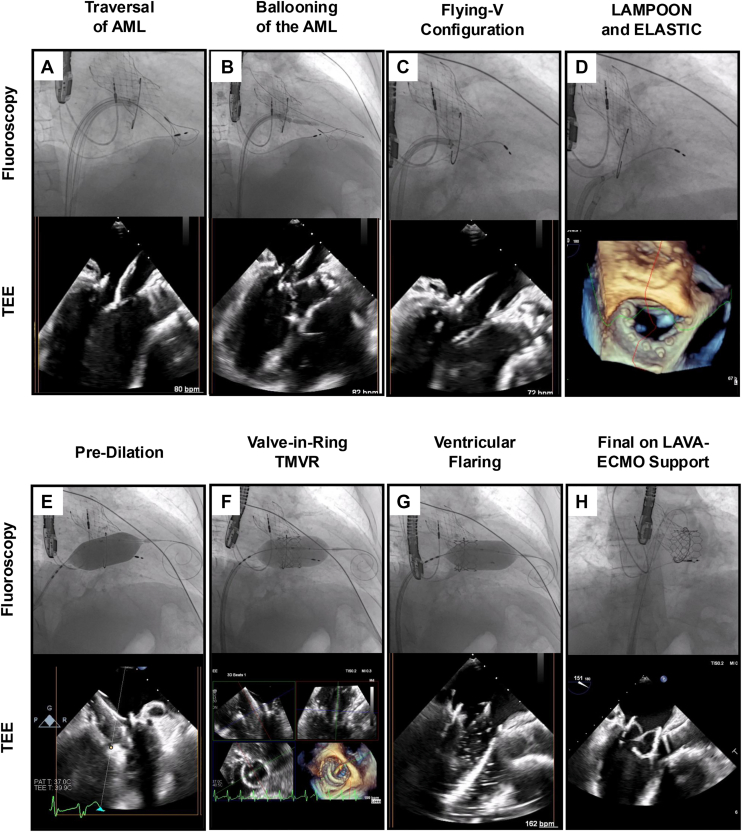
**Procedural steps on fluoroscopic and TEE imaging.** (**A**) Traversal of AML with electrified Astato wire. (**B**) Ballooning of the AML. (**C**) Flying-V configuration. (**D**) LAMPOON and ELASTIC. (**E**) Pre-dilation of the mitral valve annulus. (**F**) Valve-in-Ring TMVR. (**G**) Ventricular flaring. (**H**) Final on LAVA-ECMO support. AML, anterior mitral leaflet; ECMO, extracorporeal membrane oxygenation; ELASTIC, electrosurgical laceration of Alfieri stitch; LAMPOON, laceration of the anterior mitral valve leaflet to prevent outflow obstruction; LAVA, left atrial venoarterial; TEE, transesophageal echocardiogram; TMVR, transcatheter mitral valve replacement.

**Figure 4:**
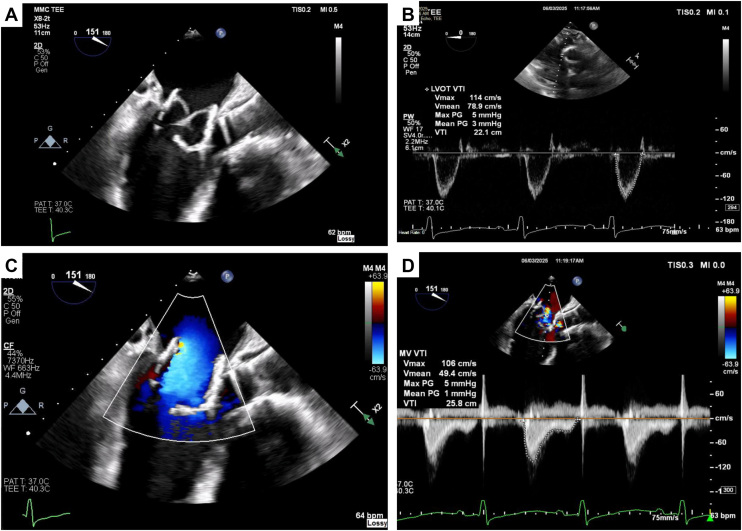
**Final transvalvular gradients.** (**A**) Valve-in-Ring TMVR on 2D TEE. (**B**) LVOT mean gradient of 3 mm Hg. (**C)** Valve-in-Ring TMVR on color TEE. (**D**) Mean transmitral valve gradient of 1 mm Hg. AML, anterior mitral leaflet; LVOT, left ventricular outflow tract; TEE, transesophageal echocardiography; TMVR, transcatheter mitral valve replacement.

This case highlights the combination of antegrade LAMPOON and ELASTIC to solve a challenging situation. Mechanical cardiocirculatory support with VA-ECMO was deemed necessary to maintain hemodynamic stability during the procedure in a patient with poor hemodynamic status at baseline.

## Declaration of competing interests

Konstantinos Koulogiannis: consultant of Edwards Lifesciences and Medtronic. Linda D. Gillam: advisor to Medtronic, Philips, and Egnite. Philippe Généreux: institutional research grants from Edwards Lifesciences; consulting fees from Abbott, Cordis, Edwards Lifesciences, Egnite, Haemonetics, Medtronic, Opsens, Puzzle Medical, Pi-Cardia, and 4C medical; equity from Puzzle Medical and Pi-Cardia; and PI of EARLY TAVR and PROGRESS trials, both sponsored by Edwards Lifesciences. Gennaro Giustino: proctor and consultant for Edwards Lifesciences, Medtronic, and Shockwave Medical and founder and shareholder of Antegrade Medical. The other authors declared no potential conflicts of interest with respect to the research, authorship, and/or publication of this article.

## Supplementary material

Supplemental Video 1 Full Procedure of Successful Valve-in-Ring TMVR Following Antegrade Base-to-Tip LAMPOON with ELASTIC on VA-ECMO Support.

Supplemental Video 2 Pre- and Post-TMVR Assessment on TEE.

## Footnotes

To access the supplementary material accompanying this article, visit the online version of the *Journal of the Society for Cardiovascular Angiography & Interventions* at 10.1016/j.jscai.2026.105394.
